# Multi-Sensor Fusion of Landsat 8 Thermal Infrared (TIR) and Panchromatic (PAN) Images

**DOI:** 10.3390/s141224425

**Published:** 2014-12-18

**Authors:** Hyung-Sup Jung, Sung-Whan Park

**Affiliations:** Department of Geoinformatics, The University of Seoul, 90 Jeonnong-dong, Dongdaemun-gu, Seoul 130-743, Korea; E-Mail: psh5759@uos.ac.kr

**Keywords:** thermal infrared (TIR), panchromatic (PAN), data fusion, Landsat 8, surface temperature

## Abstract

Data fusion is defined as the combination of data from multiple sensors such that the resulting information is better than would be possible when the sensors are used individually. The multi-sensor fusion of panchromatic (PAN) and thermal infrared (TIR) images is a good example of this data fusion. While a PAN image has higher spatial resolution, a TIR one has lower spatial resolution. In this study, we have proposed an efficient method to fuse Landsat 8 PAN and TIR images using an optimal scaling factor in order to control the trade-off between the spatial details and the thermal information. We have compared the fused images created from different scaling factors and then tested the performance of the proposed method at urban and rural test areas. The test results show that the proposed method merges the spatial resolution of PAN image and the temperature information of TIR image efficiently. The proposed method may be applied to detect lava flows of volcanic activity, radioactive exposure of nuclear power plants, and surface temperature change with respect to land-use change.

## 1. Introduction

Over the past two decades, much attention has been paid to multi-sensor data fusion for remote sensing applications such as Earth surface displacement measurements via the fusion of X-, C- and L-band SAR (synthetic aperture radar) images [1], image classification through the fusion of optic and SAR images [2,3], feature extraction with the fusion of Lidar data and optical image [4], soil moisture retrieval by the integrated use of MODIS and advanced microwave scanning radiometer (AMSR-E) data [5], vegetation monitoring from the fusion of optic and SAR images [6,7], *etc*. Data fusion is defined as the combination of data from multiple sensors such that the resulting information is better than would be possible when the sensors are used individually. One of multi-sensor advantages is an improved observability. Broadening the baseline of physical observables can result in remarkable improvements [8,9].

For example, the data fusion of panchromatic (PAN) and multi-spectral (MS) images has been widely used to create fused images with high spatial and spectral resolutions [10–12]. The data fusion of PAN and thermal infrared (TIR) images is also a good example of improved observability. The PAN image observes a radiation reflected from Earth's surface over a visible and near-infrared (NIR) wavelength range of 0.4 to 2.5 μm, whereas the TIR image observes radiation emitted from Earth's surface over a TIR wavelength range of 8 to 15 μm [13]. Thus, the brightness value of the PAN image corresponds to Earth's surface reflectance, while that of the TIR image corresponds to Earth's surface temperature. It indicates that there can be possible resulting limitations on the fusion of the PAN and TIR images because the physical processes in the PAN and TIR images are different. Nevertheless, the fusion of the remote sensed PAN and TIR images can enhance the spatial details of the TIR image and add the spatial distribution of surface temperature to the PAN image. That is, the fusion is allowed to add the thermal information of objects near the Earth's surface. To improve a TIR image at night, the edges of PAN images recorded during the daytime have been used [14] and a physical property-based post-correction solution has been proposed for the fusion of geostationary satellite TIR and visible images [15].

Landsat 8 is an Earth observation satellite launched on 11 February 2013. It is equipped with a two-sensor payload, operational land imager (OLI) and thermal infrared sensor (TIRS) [16]. In order to improve on past Landsat sensors, both of the OLI and TIRS instruments use pushbroom sensors (along-track scanner) instead of whiskbroom sensors (across-track scanner) that were utilized on earlier Landsat satellites [16]. The OLI collects data from nine spectral bands. Seven of the nine bands are similar to Landsat thematic mapper (TM) and enhanced thematic mapper plus (ETM+), but the two new spectral bands are provided: a deep blue coastal/aerosol band and a shortwave-infrared cirrus band. The PAN band (band 8) is collected with the spatial resolution of 15 m unlike other bands with the spatial resolution of 30 m. The TIRS collects two thermal infrared bands with the spatial resolution of 100 m [13]. Its application is limited by lower spatial resolution for the thermal image compared with the PAN image. Thus, the fusion of Landsat 8 PAN and TIR images must be necessary to improve the object recognition of the TIR images and add the thermal information of objects to the PAN image. Previous methods have been proposed to fuse the PAN and TIR images of Landsat 5 TM [17,18]. Since these methods limitedly focus on enhancing the spatial resolution of Landsat 5 TIR images for terrestrial Earth observation applications such as urban classification and target detection, they have added a good amount of spatial details to the TIR images, but not preserved thermal information of the TIR images. Moreover, the pixel block intensity modulation [17,18] may cause rectangular pseudo-edges because a block averaging approach is used for the fusion.

In this study, we have proposed an efficient method to fuse Landsat 8 PAN and TIR images. The fusion technique allowed us: (i) to improve the object recognition of the TIR image without losing spatial details of the PAN image and (ii) to add the thermal information of objects to the PAN image while maintaining the thermal information of the TIR image. In addition, we have introduced a scaling factor to control the trade-off between the spatial details and the thermal information. The change of the fused image with respect to the scaling factor has been tested. Finally, we have shown the performance of the fusion using Landsat 8 PAN and TIR images obtained from both urban and rural areas.

## 2. Method

The data fusion of panchromatic (PAN) and thermal infrared (TIR) images is allowed to increase the physical observables of remote-sensed images. The PAN image observes a radiation reflected from Earth's surface over the visible and NIR regions while the TIR image observes a radiation emitted from Earth's surface over the TIR region. The PAN image presents surface reflectance with high spatial resolution, whereas the TIR image shows surface temperature with low spatial resolution. Thus, the fusion of the remote sensed PAN and TIR images can be performed by combining high-passed PAN image and low-resolution TIR image. The PAN image can be decomposed into a high-passed (HP) image and a low-passed (LP) one as given by:

(1) $$I_{\text{PAN}}(i,j)=I_{\text{PAN}}^{LP}(i,j)+I_{\text{PAN}}^{HP}(i,j),$$

where *I_PAN_*(*i*,*j*) is the PAN image brightness at line *i* and pixel *j*, and $I_{\text{PAN}}^{LP}(i,j)$ and $I_{\text{PAN}}^{HP}(i,j)$ are respectively the brightness values of the LP and HP PAN images. The fusion of the PAN and TIR images can be performed by merging TIR and HP PAN images as:

(2) $$I_{\text{TIR}}^{\text{FUS}}(i,j)={\bar{I}}_{\text{TIR}}(i,j)+\alpha \cdot {\bar{I}}_{\text{PAN}}^{HP}(i,j),$$

where $I_{\text{TIR}}^{\text{FUS}}(i,j)$ is the fused TIR image brightness, *Ī_TIR_*(*i*, *j*) and ${\bar{I}}_{\text{PAN}}^{HP}(i,j)$ are the modified TIR and HP PAN image, respectively, and *α* is a scaling factor between the modified HP PAN and TIR images. The modified HP image can be defined by:

(3) $${\bar{I}}_{\text{PAN}}^{HP}=\{\begin{array}{ll} -T_{c}\cdot \sigma_{\text{PAN}}^{HP}, & I_{\text{PAN}}^{HP}\leq \mu_{\text{PAN}}^{HP}-T_{c}\cdot \sigma_{\text{PAN}}^{HP}\\ +T_{c}\cdot \sigma_{\text{PAN}}^{HP}, & I_{\text{PAN}}^{HP}\geq \mu_{\text{PAN}}^{HP}+T_{c}\cdot \sigma_{\text{PAN}}^{HP}\\ I_{\text{PAN}}^{HP}, & \text{Others} \end{array}\;,$$

where $\mu_{\text{PAN}}^{HP}$ and $\sigma_{\text{PAN}}^{HP}$ are the mean and standard deviation of a HP PAN image each and *T_c_* is a threshold value to exclude the brightest and darkest targets in the HP PAN image. The threshold value can be determined via using standard score (Z-score) given by statistical confidence level. For instance, if 95% confidence level is used, the Z-score will be 1.96, and hence the threshold value will be also 1.96, and if 99% confidence level is selected, the threshold will be 2.58. It leads to decreasing the standard deviation of the HP PAN image, but preserves spatial details in the fused image. And the modified TIR image can be also defined by:

(4) $${\bar{I}}_{\text{TIR}}(i,j)=\frac{\sigma_{\text{PAN}}^{LP}}{\sigma_{\text{TIR}}}\cdot \left(I_{\text{TIR}}(i,j)-\mu_{\text{TIR}}\right)+\mu_{\text{PAN}}^{LP}\;,$$

where $\mu_{\text{PAN}}^{LP}$ and $\sigma_{\text{PAN}}^{LP}$ are the mean and standard deviation of a LP PAN image each and *μ_TIR_* and *σ_TIR_* are respectively the mean and standard deviation of a TIR image. The scaling factor (*α*) controls the trade-off between the spatial details and the thermal information. If *α* = 1, the fused image has a similar statistical property of the PAN image. The thermal information is easy to notice when *α* is close from 1 to 0, whereas the spatial details stand out if *α* is more than 1. The optimal value of the scaling factor can be calculated as given by:

(5) $$\alpha =\frac{\sqrt{\sum_{i=1+w}^{N-w}\sum_{j=1+w}^{M-w}{\left\{{\bar{s}}_{\text{TIR}}(i,j)\right\}}^{2}}}{\sqrt{\sum_{i=1+w}^{N-w}\sum_{j=1+w}^{M-w}{\left\{{\bar{s}}_{\text{PAN}}^{HP}(i,j)\right\}}^{2}}}\;,$$

where *N* and *M* are the numbers of lines and pixels in the modified HP PAN and TIR images, respectively, *w* is the half of moving-window size, and ${\bar{s}}_{\text{PAN}}^{HP}$ and *s̄_TIR_* are the standard deviations of the samples extracted using moving-window in the modified HP PAN and TIR images each. The optimal value gets the root-mean-squares (RMS) of the local standard deviations of the modified HP PAN and TIR images ( ${\bar{s}}_{\text{PAN}}^{HP}$ and *s̄_TIR_*) to be identical. To wit, the range of brightness values in the HP PAN image becomes very similar to that of brightness values in the TIR image.

**Figure 1.**
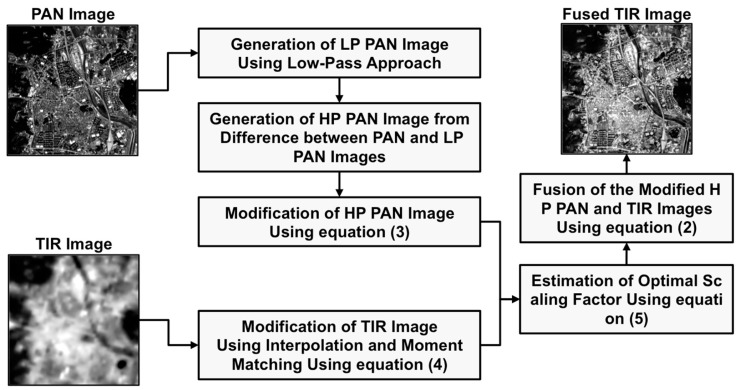
Detailed workflow of the proposed method, where the PAN and TIR stand for panchromatic and thermal infrared, respectively.

Consequently, the optimal value enables us to fuse the HP PAN and TIR images at a similar brightness level, and at the same time to recognize the spatial variation of the surface temperature and the morphological and positional information of the objects from the fuse image. The proposed processing method is summarized in Figure 1 and composed of six steps as follows:

(1) Generation of a LP PAN image using a low-pass approach from a PAN image, where the low-pass approach is implemented by block averaging and then interpolation;
(2) Generation of a HP PAN image by subtracting the LP PAN image from the PAN image;
(3) Modification of the HP PAN image using Equation (3), which is used for the removal of highest and lowest brightness values;
(4) Modification of a TIR image by interpolation and then moment matching using Equation (4) from the mean and standard deviation of the LP PAN image;
(5) Estimation of optimal scaling factor by calculating the root-mean-squares of local standard deviations of the modified HP PAN and TIR images using Equation (5), and
(6) Creation of a fused image from the modified HP PAN and TIR images using Equation (2).

The image fused by the proposed method allows for the preservation of the surface temperature information and the spatial resolution. In this study, we have estimated the optimal scaling factor using Equation (5), but the optimal value may be changed in some application fields of remote sensing. For example, military applications of remote sensing require more spatial details, and then the scaling factor can be increased.

## 3. Results and Discussion

The proposed fusion approach using Landsat 8 PAN and TIR images has been tested. The test data was acquired on 30 May 2014 from 116/034 (path/row) in the world reference system-2 (WRS-2). The test data covers Seoul in South Korea and Kaesong in North Korea including high mountains and agricultural fields in Kaesong and metropolitan areas in Seoul. Consequently, we have tested the performance of the fusion approach in both urban and rural areas.

**Figure 2.**
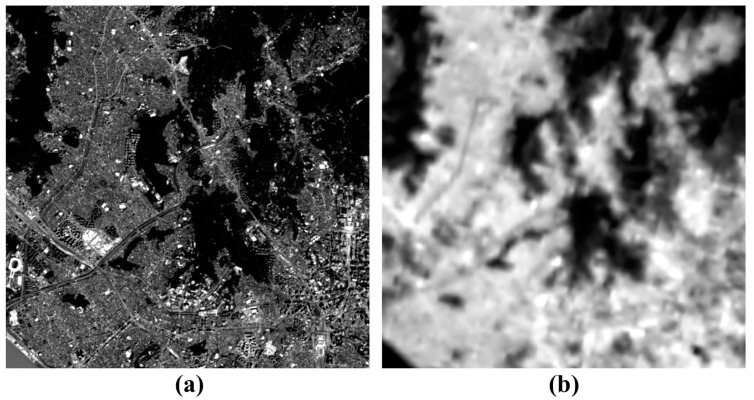
(**a**) PAN and (**b**) TIR images of Landsat 8 used for the fusion test.

Figure 2a,b presents Landsat 8 PAN and TIR images, respectively. Landsat 8 was launched on 11 February 2013. Landsat 8 acquires a PAN image with the spatial resolution of 15 m and TIR images with the spatial resolution of 100 m, and hence the PAN image has about seven times higher spatial resolution than the TIR images. We can see spatial details, which include buildings, roads, and the like from the PAN image of Figure 2a, but we cannot recognize them from the TIR image of Figure 2b. As seen in the PAN image (Figure 2a), most mountains and rivers have relatively lower reflectance values, and most urban areas are brighter than the mountains and rivers but darker than brightest targets such as tall buildings. As for the TIR image, most urban areas are much brighter than the mountains and rivers (Figure 2b). It should be noted that ‘bright’ means ‘warm’ in the TIR image while ‘dark’ means ‘cold’. And the brightest target positions in the TIR image are different from those in the PAN image, because the TIR image is not related to the surface reflectance, but with the surface emittance. That is, the brightness value of the PAN image is correspondent to the surface reflectivity while that of the TIR image is related to the surface temperature.

The fusion of the PAN and TIR images is accomplished by mixing the surface reflectivity and temperature. Thus, the fusion is required to be carefully performed because the two different properties of the ground surface are fused efficiently and properly. If the proper fusion approach is applied, it will make us to simultaneously interpret the spatial variation of the surface temperature and the morphological and positional information of objects. Otherwise, we don't need to fuse the PAN and TIR images at all.

We have tried to fuse the PAN and TIR images of Figure 2a,b. We have generated an LP PAN image using a low-pass approach from the PAN image. The low-pass approach was applied in the spatial domain rather than the frequency domain. To consider the spatial resolution and interpolation effect of the TIR image, the low-pass approach is done by two steps of: (i) block-averaging using the window size of 7 × 7 pixels and (ii) interpolating the block-averaged image to the original image size using the bicubic interpolation method. An HP PAN image was generated by subtracting the LP PAN image from the original PAN image. Then, the data range of the HP PAN image was reduced from the Equation (3) using the threshold value of 1.96 corresponding to the confidence level of 95%. The TIR image was interpolated to the original PAN image size using the bicubic interpolation method, and then converted into the mean and standard deviation of the LP PAN image using the moment matching method of Equation (4). The fused image was created using Equation (2) by summation of the modified TIR image and the HP PAN image scaled by *α*. In this test, we haven't estimated the optimal scaling factor using Equation (5) to evaluate the fusion performance according to the scaling factor.

**Figure 3.**
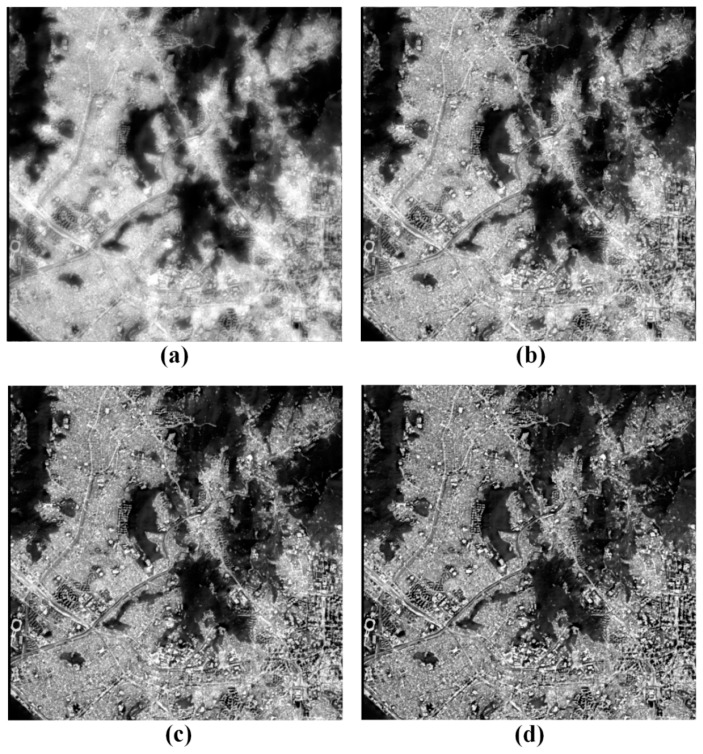
The fused images from the PAN and TIR images of Figure 2a,b by using the scaling factors of (**a**) *α* = 0.25; (**b**) *α* = 0.5; (**c**) *α* = 0.75 and (**d**) *α* = 1.0.

Figure 3 compares the images fused from the different scaling factors. The fused images from Figure 3a–d were generated using the scaling factors of 0.25, 0.5, 0.75 and 1.0. As aforementioned, the scaling factor is used to control the trade-off between the spatial details and thermal information. The higher the scaling factor is, the higher the spatial resolution is. On the other hand, the thermal information gets to lose its details. The spatial distribution of surface temperatures is steadily distorted as the scaling factor gets higher (Figure 3). When compared with the TIR image of Figure 2b, the fused image of *α* = 0.25 (Figure 3a) preserves the spatial variation of the surface temperature well while the fused image of *α* = 1.0 (Figure 3d) loses the spatial distribution of the temperature difference, especially in the urban area. The morphological and positional information of objects gets more noticeable as the scaling factor is higher (Figure 3). When compared with the PAN image of Figure 2a, the fused image of *α* = 0.25 (Figure 3a) shows low distinguishability while the fused image of *α* = 1.0 (Figure 3d) displays high distinguishability. The trade-off between the thermal information and distinguishability is very clear as shown in Figure 3.

First and foremost, the fusion of the TIR and PAN images enables us to analyze the spatial variation of the thermal information among objects near the surface of the Earth from the fused image. We would have difficulties in classifying the objects near the Earth's surface from the fused image of Figure 3a and in analyzing the spatial distribution of the temperature difference from the fused image of Figure 3d. On the other hand, we can see that the fused images of Figure 3b,c allow us to simultaneously recognize both the spatial variation of the surface temperature and the morphological and positional information of the objects in the fused image.

The optimal scaling factor can be determined by the minimum value among the scaling factors that make the objects in the fused image distinguishable. Since we can identify as many objects from the fused image of Figure 3b as we can from that of Figure 3c, the fusion with *α* = 0.5 might be better than *α* = 0.75 because its thermal information evidently gets clearer as seen in Figure 3b,c. For that reason, the scaling factor of about 0.5 can be selected as an optimal value with the caveat that this evaluation may be arguably subjective rather than objective. Thus, an automatic approach for optimal parameter estimation is required to improve the fusion performance.

**Figure 4.**
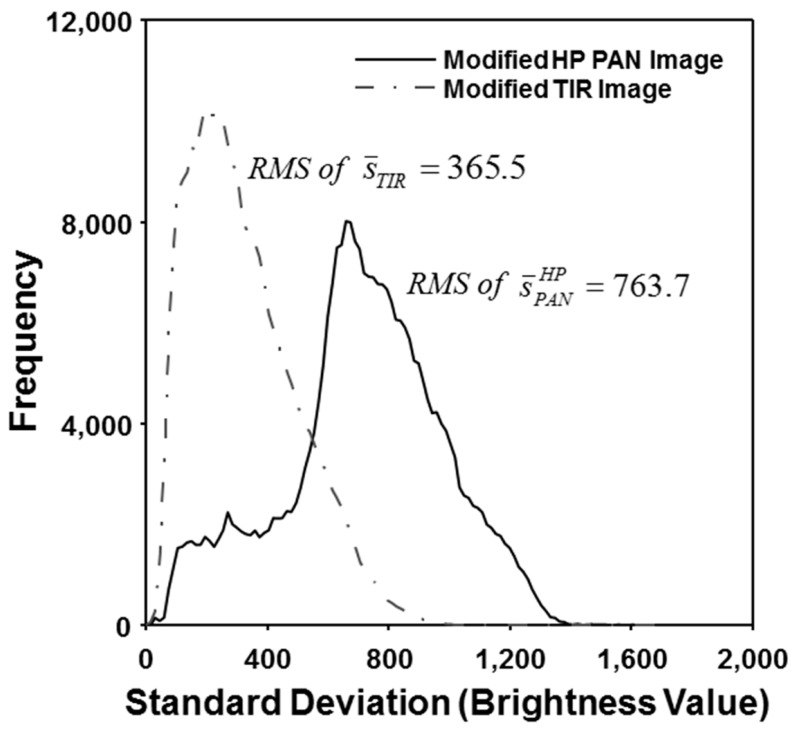
Histograms of local standard deviations calculated by using the window kernel of 21 × 21 from the modified HP PAN and the modified TIR images.

The optimal parameter estimation exploits that there are an approximate relation between the local standard deviation and the object identification. Based on this relation, the estimation process is implemented by equalizing the RMS of the local standard deviations calculated from the modified HP PAN and TIR images. Figure 4 shows the histograms of the local standard deviations derived from the two images using the moving window of 21 × 21. As seen in Figure 4, the local standard deviations of the modified HP PAN image are higher than those of the modified TIR image. The RMS of the local standard deviations of the modified HP PAN image was 763.7, whereas that of the modified TIR image was 365.5. The optimal scaling factor was determined from the ratio between the two RMS of the local standard deviations, which was about 0.48. It means that the local brightness range of the modified HP PAN image becomes very close to the modified TIR image. That is, because the modified HP PAN and TIR images can be fused at the same brightness level, we can identify the spatial variation of the surface temperature as well as spatial details of the objects from the fused image.

**Figure 5.**
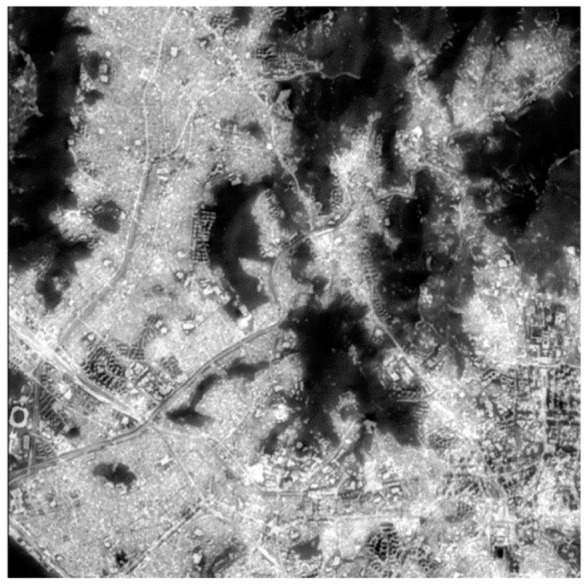
The fused image from the PAN and TIR images of Figure 2a,b using the optimal scaling factor estimated by Equation (5).

Figure 5 shows the image fused by using the optimal parameter of about 0.48, which was estimated by Equation (5). We cannot identify any buildings and roads from the TIR image of Figure 2b, while we can clearly see the buildings and roads in the fused image of Figure 5. We can also recognize that the mountainous area has a lower temperature than the urban area from the fused image. Moreover, we can identify high and low temperature areas in urban area and interpret this like tall buildings are in the low temperature areas from the fused image. However, although the roads are brighter than the adjacent buildings, we cannot say that the temperature of the roads is higher than that of the nearby buildings. That is because the brightness difference does not indicate temperature difference but reflectance difference. Therefore, the proposed fusion method has a disadvantage that an intuitive interpretation of the fused image is very difficult, because two different spatial information is mixed. It should be noted that high spatial frequencies show the reflectance information in the fused image while low spatial frequencies present the temperature information.

**Figure 6.**
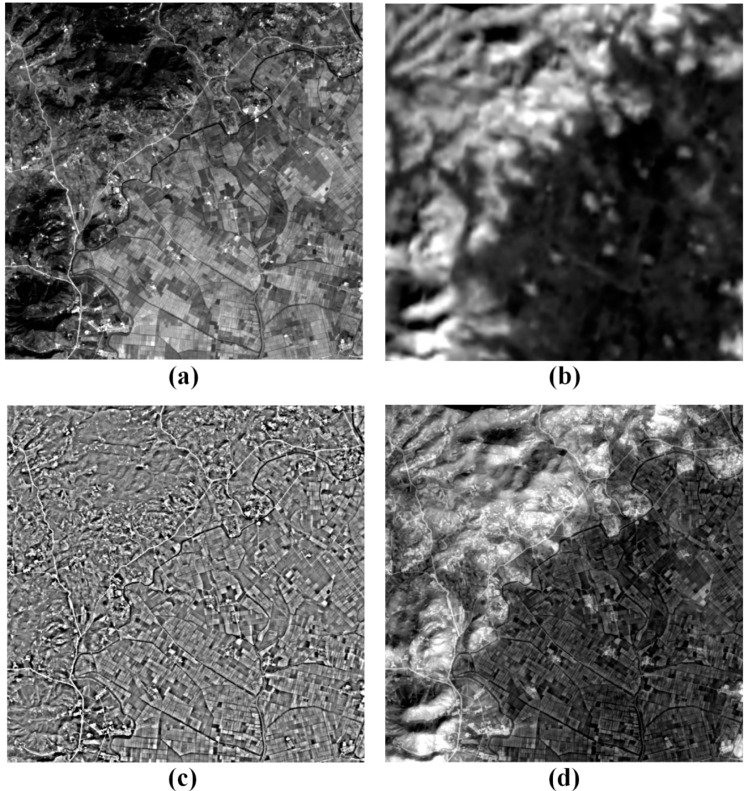
Fused result of the PAN and TIR images obtained from rural areas: (**a**) the PAN and (**b**) TIR images; (**c**) the HP PAN image and (**d**) the fused image.

Figure 6 shows the fused result of the PAN and TIR images obtained from rural areas. As seen in Figure 6a, there are mountains in the left part of the image and rice fields in the right part of the image. In the PAN image, the brightest objects are most of the artificial targets such as roads and buildings, and the darkest ones include lakes and mountains. The rest is most of the rice fields. On the other hand, in the TIR image, the brightest objects are mountains while the darkest ones are rice fields (Figure 6b). We cannot recognize the artificial targets in the TIR image due to its low spatial resolution. We can see that the most brightness values of the PAN image are inversely proportional to those of the TIR image in Figure 6a,b.

The HP PAN image of Figure 6c highlights the edges and boundaries of the buildings, roads, rice fields, and the like. It is used to enhance the distinguishability of the objects in the TIR image. Figure 6d is the fused image created by combining the PAN and TIR images. The estimated optimal scaling factor was about 0.77. The optimal value is much higher than that of about 0.48 used for the fusion of Figure 2a,b. That is because the RMS of the local standard deviations of the HP PAN image shown in Figure 6c were much lower than that of the HP PAN image used for the fusion of Figure 2a,b. It indicates that the reflectance difference of urban area is larger than that of rural area.

In the fused image, mountains are displayed by the brightest areas while rice fields are shown by the darkest areas. This means that the low spatial frequency brightness pattern of the fused image is very similar to that of the TIR image. Moreover, we can identify the spatial details of the objects including the roads, buildings, rivers and lakes from the fused image. If we can only use the PAN image of Figure 6a, we cannot tell that the mountains have been deforested, but the fused image of Figure 6d enables us to know the mountain deforestation because the brightness values, that is, temperature, of the mountains are higher than those of the rice fields. The result reveals that we can extract the temperature information and spatial details of the objects from the fused image simultaneously.

**Figure 7.**
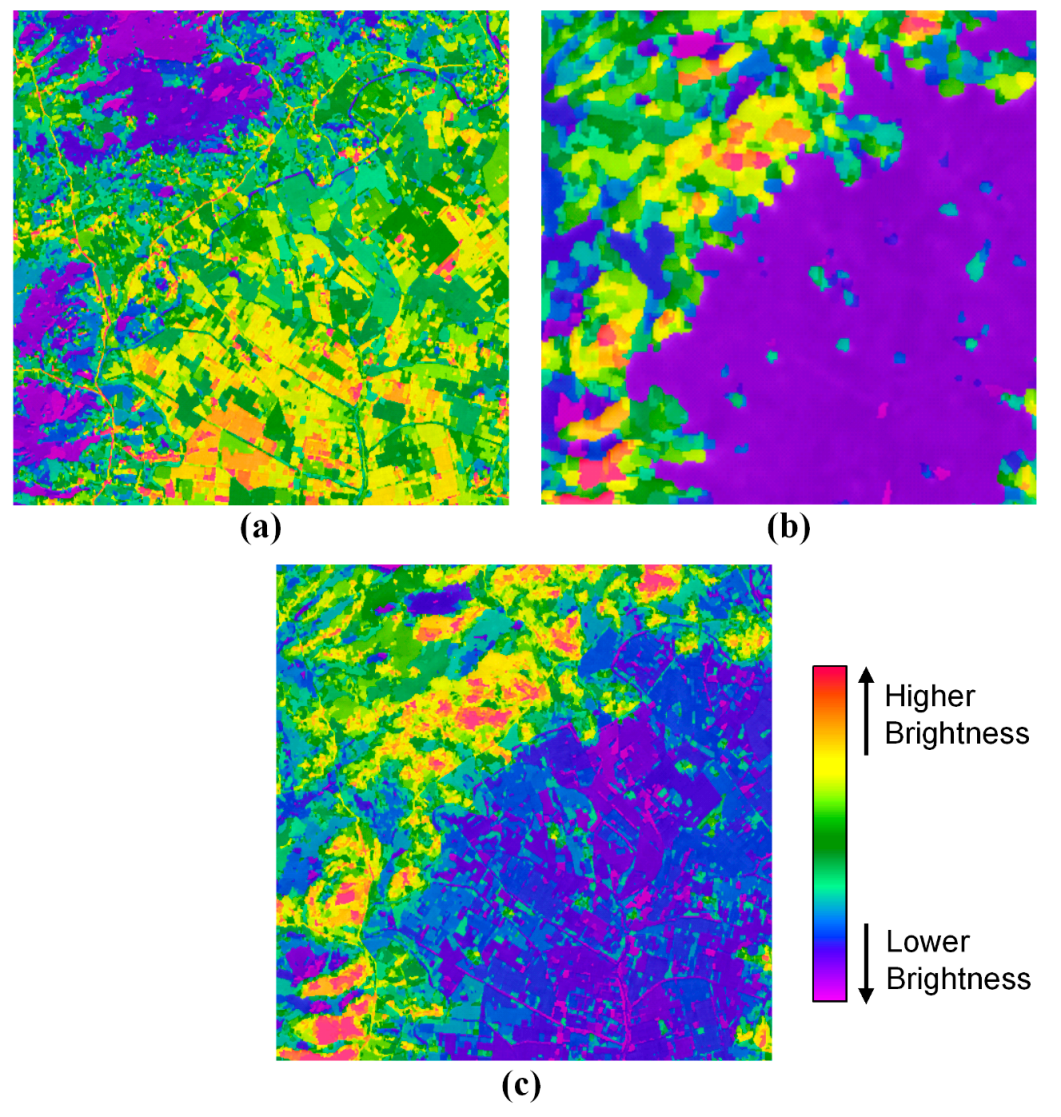
Image segmentation results from (**a**) the PAN image; (**b**) the TIR image and (**c**) the fused image of Figure 6 using the watershed transform.

To evaluate the fusion performance of the proposed method, we carried out the image segmentation using the watershed transform [19]. The image segmentation was tested under the same conditions. Figure 7 shows the segmentation results from the PAN, TIR and fused images of Figure 6. Comparing the PAN segmentation image of Figure 7a with the fused segmentation image of Figure 7c, the number of segments was similar, but the brightness pattern was different.

While the fused segmentation image was larger at the number of segments than the TIR segmentation image, the brightness pattern between the fused and TIR segmentation images is very similar. It means that the fused image by the proposed method is similar at the spatial resolution to the PAN image and at the spatial pattern of the brightness values to the TIR image. Therefore, the result proves that the proposed method can fuse PAN and TIR images efficiently and successfully.

**Figure 8.**
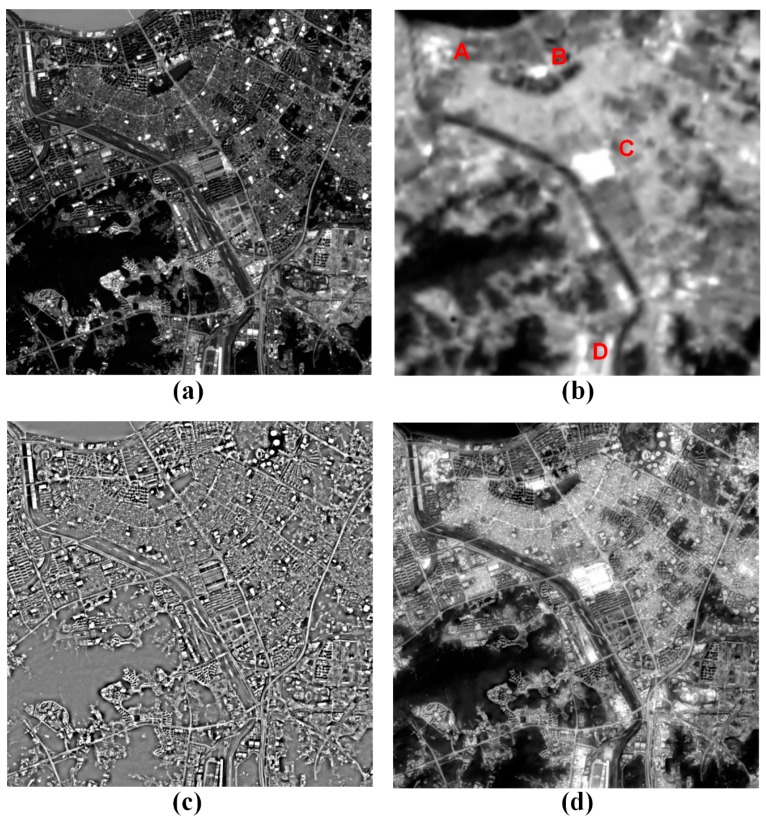
Fused result of the PAN and TIR images obtained from urban areas: (**a**) the PAN and (**b**) TIR images; (**c**) the HP PAN image and (**d**) the fused image.

Figure 8 shows the fused result of the PAN and TIR images obtained from urban areas. As shown in Figure 8a, there are a lot of buildings and roads. In the PAN image, the brightest objects are most of the tall buildings, and the darkest ones include mountains and parks. The rest is most of the buildings, roads, river, and the like. In the TIR image, the darkest objects are most of the mountains and river as seen in Figure 8b. It seems to display a similar pattern of the PAN image. However, the brightest objects of the TIR image are quite different from those of the PAN image. For instance, in the TIR image, we can find the brightest objects in the areas of A, B, C and D.

We have difficulties in identifying the objects due to the low spatial resolution of the TIR image. However, we can recognize the objects from the fused image of Figure 8d because the spatial details were improved. The fusion was processed using the optimal scaling factor of about 0.57. The hot spots of A, B, C and D are respectively a sport complex, an amusement park, a fish market and an airport. The most hot spots are crowded places. One of the reasons why most crowded places might be much warmer than other ones may be they are high-energy consumption places. Moreover, we can analyze the spatial distribution of the surface temperature according to the type of the residence from the fused image. The residential density strongly links to the surface temperature. As seen in Figure 8d, we can find that the surface temperatures in the high-rise areas are lower than those in the highest-density housing. Thus, the fused image is allowed to distinguish the difference of the surface temperature among the objects near the Earth's surface.

**Figure 9.**
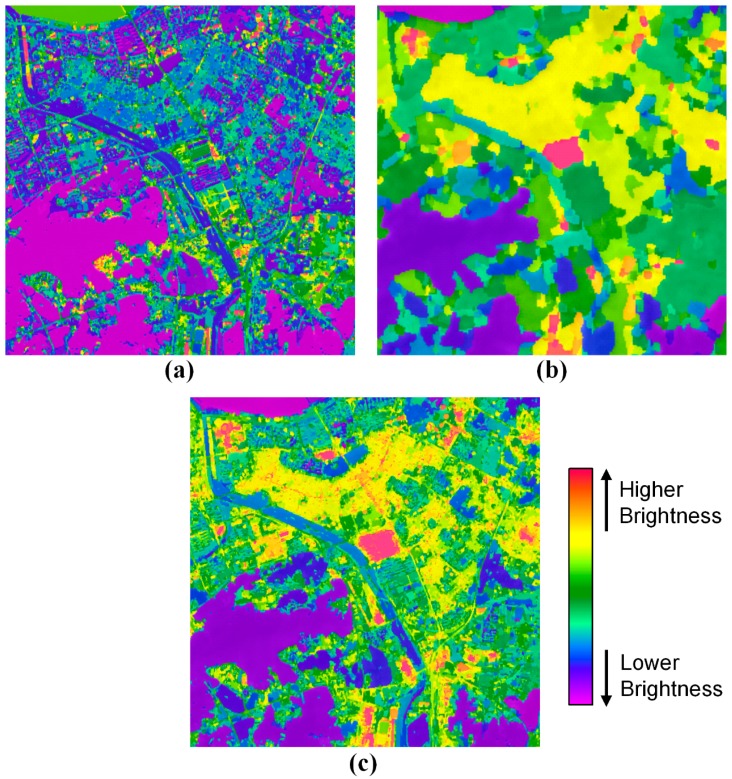
Image segmentation results from (**a**) the PAN image; (**b**) the TIR image and (**c**) the fused image of Figure 8 using the watershed transform.

The image segmentation analysis was tested under same condition for the PAN, TIR and fused images of Figure 8. Figure 9 shows the segmentation results. The fused segmentation image is similar to the PAN image at the spatial resolution and to the TIR image at the spatial pattern of the brightness values. The result shows that the fusion of PAN and TIR images though our proposed method merges the spatial resolution of PAN image and the temperature information of TIR image both efficiently and successfully.

The proposed method can be applied to detect lava flows of volcanic activity, radioactive exposure of a nuclear power plant, the surface temperature change with respect to land-use change, *etc*. And it is also used for merging: (1) the VNIR (visible and near-infrared) and TIR images of ASTER or MODIS data products; (2) the PAN and MIR (middle-infrared) images of Kompsat-3A, *etc*. Especially for Kompsat-3A, the proposed method can be used for high-resolution image fusion product. In addition, the proposed method can also be used for the fusion of high-resolution SAR and low-resolution TIR images as well as the fusion of high- and low-resolution optic images.

## 4. Conclusions

The multi-sensor fusion of PAN and TIR images is a good example of an improved observability. The PAN image observes a radiation reflected from the Earth's surface over a visible and near-infrared (NIR) wavelength range of 0.4 to 2.5 μm, while the TIR image observes a radiation emitted from Earth's surface over a TIR wavelength range of 8 to 15 μm. According to the amount of radiant energy, the PAN image has high spatial resolution while the TIR image has low spatial resolution. Thus, the fusion allows one: (i) to improve the object recognition of the TIR image while preserving spatial details of the PAN image and (ii) to add the thermal information of objects to the PAN image while maintaining the thermal information of the TIR image.

In this study, we have proposed an efficient fusion method to fuse Landsat 8 PAN and TIR images. The method is designed to preserve the surface temperature information as well as the spatial resolution. We have introduced a scaling factor to control the trade-off between the spatial details and the thermal information. The method has been tested using Landsat 8 PAN and TIR images. The test sites cover Seoul in South Korea and Kaesong in North Korea including high mountains and agricultural fields in Kaesong and metropolitan areas in Seoul. The change of the fused image with respect to the scaling factor (*α*) has been tested. The fused image with *α* = 0.25 preserves the spatial variation of the surface temperature well while it shows low distinguishability, and the fused image with *α* = 1.0 does not keep the spatial distribution of the temperature difference well while it displays high distinguishability. We can simultaneously recognize not only the spatial variation of the surface temperature, but also the morphological and positional information of the objects in the fused images with *α* = 0.5 and 0.75. If you want more specific thermal information, the choice of *α* = 0.5 might be better. Second, we have tested the fusion of the PAN and TIR images obtained from rural areas. From the fused results, we could recognize the mountain deforestation from the fact that the temperature of mountains is higher than that of the rice fields. Finally, we have tested the fusion in urban areas. We could analyze the spatial distribution of the high-energy consumption places as well as the type of the residence from the fused image.

The proposed fusion method is readily expected to be applicable to detect lava flows of volcanic activity, radioactive exposure of a nuclear power plant, the surface temperature change with respect to land-use change, *etc*. Moreover, the method can also be used for fusing: (1) the VNIR (visible and near-infrared) and TIR images of ASTER or MODIS data products; (2) the PAN and MIR (middle-infrared) images of Kompsat-3A.
